# GAPCNN with HyPar: Global Average Pooling convolutional neural network with novel NNLU activation function and HYBRID parallelism

**DOI:** 10.3389/fncom.2022.1004988

**Published:** 2022-11-15

**Authors:** Gousia Habib, Shaima Qureshi

**Affiliations:** Department of Computer Science and Technology, National Institute of Technology Srinagar, Srinagar, India

**Keywords:** Global Average Pooling, NNLU, CNN, AMsgrad, SGD, ADAM, hybrid parallelism, max-pooling

## Abstract

With the increasing demand for deep learning in the last few years, CNNs have been widely used in many applications and have gained interest in classification, regression, and image recognition tasks. The training of these deep neural networks is compute-intensive and takes days or even weeks to train the model from scratch. The compute-intensive nature of these deep neural networks sometimes limits the practical implementation of CNNs in real-time applications. Therefore, the computational speedup in these networks is of utmost importance, which generates interest in CNN training acceleration. Much research is going on to meet the computational requirement and make it feasible for real-time applications. Because of its simplicity, data parallelism is used primarily, but it performs badly sometimes. In most cases, researchers prefer model parallelism to data parallelism, but it is not always the best choice. Therefore, in this study, we implement a hybrid of both data and model parallelism to improve the computational speed without compromising accuracy. There is only a 1.5% accuracy drop in our proposed study with an increased speed up of 3.62X. Also, a novel activation function Normalized Non-linear Activation Unit NNLU is proposed to introduce non-linearity in the model. The activation unit is non-saturated and helps avoid the model's over-fitting. The activation unit is free from the vanishing gradient problem. Also, the fully connected layer in the proposed CNN model is replaced by the Global Average Pooling layers (GAP) to enhance the model's accuracy and computational performance. When tested on a bio-medical image dataset, the model achieves an accuracy of 98.89% and requires a training time of only 1 s. The model categorizes medical images into different categories of glioma, meningioma, and pituitary tumor. The model is compared with existing state-of-art techniques, and it is observed that the proposed model outperforms others in classification accuracy and computational speed. Also, results are observed for different optimizers', different learning rates, and various epoch numbers.

## 1. Introduction

Training neural networks are time-consuming and labor-intensive, restricting the use of deep learning systems in various real-time applications. Thus, there is a requirement for faster learning, particularly when considering CNN parallelization. Stochastic gradient descent (SGD) and its variants are frequently employed for deep learning model training. Nevertheless, the major issue of SGD is that it spreads the gradient information uniformly in every direction, which is not appropriate for use in applications with impoverished gradient scaling. In these circumstances, it is very difficult to control the learning rate α, while training to ensure that it eventually reaches the local minimum.

**Figure 1:**
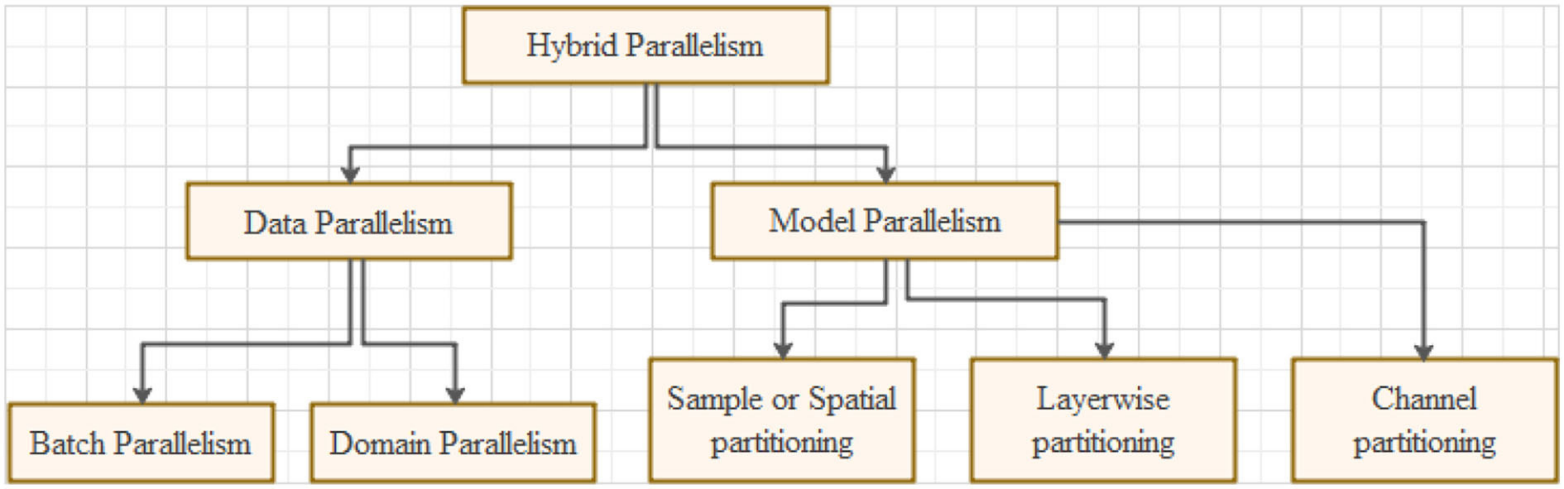
Parallelization hierarchy.

Changing the decay rate is complex and it depends on the dataset. Convolutional neural networks have made significant progress recently in object classification and detection (Ren et al., 2015; Han et al., 2018), image classification (Haralick et al., 1973; Kim et al., 2008), texture classification (Huang et al., 2014; Pang et al., 2017), hand gesture recognition (Stanescu et al., 2016; Yang et al., 2018), bio-medical image analysis (Huang et al., 2014; Pang et al., 2017), speech recognition (Stanescu et al., 2016; Yang et al., 2018), natural language processing (Stanescu et al., 2016; Yang et al., 2018), and other sophisticated deep learning models. These models necessitate a large quantity of data for the training, prompting considerable computation. To reduce the huge computation requirements, distributed systems and clusters are commonly used to make the training process more parallel. Data parallelization and model parallelization are the primary techniques to achieve parallelization. The graphical representation of the training approach hierarchy shown in Figure 1 is the best way to visualize it.

Various parallelization methods are data parallelism, batch parallelism, domain parallelism, model parallelism, spatial partitioning, channel filter partitioning, layer-wise portioning, and hybrid parallelization. These methods are broadly elaborated in Habib and Qureshi (2020b). The current paper is divided into different sections. Section 2 describes the main contributions of the study. Section 3 provides the related study. Sections 5 and 6 provide experimental details, results, and discussion. Section 7 concludes the paper. To the best of our knowledge, this is the first paper implementing hybrid parallelism with novel activation function NNLU and GAP layer for detection and classification of bio-medical images.

## 2. Main contributions of the study

### 2.1. Motivation behind novel activation function

#### 2.1.1. Challenges

The activation function of a CNN introduces a desired non-linearity into the network. Non-linearities introduced between consecutive layers can generate a more expressive model, which is ideal for deep networks. The output should not be a simple linear combination of the input to have discriminating power encoded into weights of the convolutional kernels. To avoid the vanishing gradient problem during gradient descent training of deep neural networks, a function lacking sign symmetry (anti-symmetric) is used which gives only positive output values. The error flow through back-propagation might drop exponentially when applying the sigmoid activation function till it vanishes. This can be mitigated by using derivatives of positive values. The most common activation function in the convolutional layer is the Rectified Linear Unit or ReLu. The error flow through back-propagation might drop exponentially when applying the sigmoid activation function till it vanishes. This can be mitigated by using derivatives of positive values.

#### 2.1.2. Suggested solution

To avoid the vanishing gradients problem during gradient descent training of deep neural networks, an anti-symmetric function that gives only positive output values is used. The proposed novel activation function is taken from the derivation of the diffusion equation based upon some essential properties such as normalized and unsaturated functions showing improvement over the most popular ReLu activation function. A detailed description of the function is given in the next section.

### 2.2. The motivation behind introducing Global Average Pooling layer (GAP layer)

#### 2.2.1. Challenges

The feature maps of the last convolutional layers of CNN are vectorized and are given as input to fully connected layers, followed by a soft-max logistic regression layer. These vectorized convolutional structures are then combined with classic neural networks. As the fully connected layer is prone to over-fitting, dropout can be used as a regularizer, making half of the activations of the fully connected layers zero at random during training. This increases the generalization of the neural network and significantly reduces over-fitting. However, using dropout is not always a feasible solution.

#### 2.2.2. Suggested solution

The flattened layers in CNN, are replaced by Global Average Pooling layers. In the last Conv. layer, one feature map for each relevant category of the classification task is generated. Rather than adding fully connected layers on the top of feature maps, the Soft-Max layer takes the average of each feature map. Another, advantage of this approach is that there is no parameter to optimize Global Average Pooling, so over-fitting is avoided. Global Average Pooling sums out the spatial information, making it more robust for the spatial translation of inputs. It can be seen as a structural regularizer that explicitly forces feature maps to have confidence.

### 2.3. The motivation behind introducing HyPar (fusion of data and model parallelism)

#### 2.3.1. Challenges

The outstanding achievement of CNNs in computer vision, text classification, satellite imagery, and game playing, have demanded increased computational requirements for the training. These models take days even weeks to train from scratch on the latest GPUs. Many existing parallelization techniques have come across to improve the training of CNNs. Most of the techniques employ data parallelism for the training acceleration of CNNs. But this technique poses some critical challenges. The main problem with this type of parallelism is that it only works for models with fewer parameters. There is a gradual loss of performance as the parameter size increases. Furthermore, as the mini-batch size increases beyond the *N*-value, the inference accuracy of the trained neural network begins degrading, resulting in an overall network performance reduction.

Another commonly used method is model parallelism which splits the entire network into disjoint sets, and each disjoint set is allocated to the dedicated device to improve the training efficiency. The model parallelism strategy considers only intra-layer computation, assuming that data already exists in memory since it is a fine-grained parallelism strategy. Compared to data-parallel techniques, it involves more data transfer operations and incurs more computational costs. To get rid of this overhead, the underlying architecture for the deep neural system needs to be carefully designed before training starts.

#### 2.3.2. Suggested solution

A higher degree of parallelism is achieved by combining both data parallelism and model parallelism. This is known as hybrid parallelism. Due to the different complexity of various layers of CNN, the Hybrid parallelism technique partitions the CNN's so that convolutional layers and pooling layers exploit data parallelism, and fully connected layers exploit model parallelism.

## 3. Related study

The exceptional performance of CNNs in computer vision systems, text categorization, space photography, and game-playing has necessitated significant computing speedup for CNN training. These CNNs may require days or even weeks to get trained from scratch on modern processing units. Many existing parallelization approaches have already been developed to enhance CNN training. The majority of approaches use data parallelism to accelerate CNN training. However, as previously stated, the method presents some significant problems. Another widely used approach is parallel model training. The entire network is divided into disjointed sections, and each set is assigned to a dedicated device for enhanced learning.

Jiang et al. (2020) suggested a parallelization technique known as layer-wise parallelism, which allows each layer to use an independent parallelization strategy. Optimization of each layer is done by the use of graph search. They use two distinct graphs to describe the parallelization challenge properly. The device and computation graphs reflect all the existing hardware resources and interconnectivity of devices. The latter discusses the allocation of CNN networks to a device graph. The proposed cost model analyses the individual run time parallelization efficiency using the dynamic graph-based search method. This also helps in determining the optimum parallelization approach. Layer-wise parallelism outperforms existing state-of-the-art methods in terms of communication cost, throughput, and scalability with the addition of more devices (Deliège et al., 2021).

Existing solutions, such as Tensor Flow and MXNET, only focus on one parallelization approach simultaneously, necessitating a huge amount of data to scale (Wang et al., 2018). The authors suggested a method for determining the best tiling strategy for partitioning tensors while minimizing communication costs across devices. The suggested method combines data and model parallelization (SOYBEAN). The SOYBEAN method uses an automated parallelization technique. When implemented on Alex Net and VGG, the authors discovered that SOYABEAN outperforms data parallelism by 1.54 (Christlein et al., 2019).

These authors suggested a novel parallelization training approach called SOAP (Jia et al., 2019). This method employs CNN parallelization in the sample, operation, attribute, and parameter dimensions. SOAP uses the Flex Flow deep learning platform, which determines the best parallelization approach for a given system. The optimum parallelization approach predicted by this framework is three times faster than prior techniques. The suggested technique is tested on 6 deep learning models with two GPU clusters. The suggested platform improved learning efficiency by 3.8x over state-of-the-art approaches. It has also been demonstrated that the technique improves the scalability of many devices (LeCun et al., 1998).

The authors showed that model parallelism grows with the size of the mini-batch (Oyama et al., 2020). As the mini-batch size increases, the inaccuracy decreases gradually. As a result, the parallelism is severely limited by the mini-batch size. This may be mitigated by using hybrid parallelism. They also utilized a 3D CNN to predict cosmogenic variables from three-dimensional mass distribution by utilizing HyPar on 128 GPUs and making use of a sample that was 64 times the size of the original sample. Their suggested approach enables clients to employ model partitioning and spatial dimensions of samples for each CNN layer. This increases versatility and provides excellent load balancing across multiple GPUs. The authors achieve 171 TFlops training acceleration on the Cosmo Flow framework by using hybrid-parallelism on 128 Tesla V 100 GPUs (Krizhevsky et al., 2012).

With the increasing amount of data sets, it is important to train CNNs effectively with decreased learning time and large dataset scalability (Dryden et al., 2018). The use of a big dataset necessitates a significant size of memory which is one of the challenges that must be addressed. Data parallelism by default is not a viable option since it splits samples inside the mini-batch and restricts adaptability to the large size of mini-batches, enforcing high memory cost, and making real-time deployment of conv models challenging. They suggested a novel convolution technique incorporating samples and spatial tensor decomposition. The author also created a performance model for CNNs and proposed a method for determining the optimum parallelization approach. The suggested method is tested using the ResNet-50 model for image detection and classification. The authors revealed that the suggested technique outperformed prior parallelization approaches in terms of performance and provides strong scalability. Also, the method permitted training on massive inaccessible data (Simonyan and Zisserman, 2014).

A novel technique for dividing neural networks for effective execution in distributed parallel paradigms has been suggested (De Campos Jr et al., 2020). The suggested approach is responsible for optimizing the inference rate and reducing the number of communicating nodes in a neural network. They experimented with a limited number of steps using LeNet5 with increased inference rate maximization. For partitioning LeNet5, the proposed approach delivers 38% more inferences per second than the most popular partitioning frameworks, such as TensorFlow and Metis. The authors utilized nine techniques for partitioning CNN to analyze the effect of the convergence rate inside a distributed environment for resource constrained devices. DN^2^PC IoT partitions neural networks as graphs in a distributed way across numerous IoT devices to achieve maximum convergence rate and minimize intercommunication costs between different devices. The suggested method is also in charge of efficiently handling the network's memory requirements, which are shared by CNN parameters and biases. The suggested technique leads to appropriate partitioning for IoT devices. DN^2^PC IoT also made the system more versatile and enabled the inclusion of additional goal functions (Szegedy et al., 2015).

Due to limited bandwidth, memory, and power constraints, CNN accelerators struggle to provide superior quality resolution for both image and video on edge devices (Hunag et al., 2019). As a result, efficient micro-architecture in terms of computation power and memory cost for such embedded devices is critical for speeding up inference. The authors suggested the ERNet hardware-based network optimized image and video resolution depending on hardware restrictions. They developed FBISA and a coarse-grained instruction set architecture to overcome restricted power constraints. Finally, the authors proposed an embedded CNN processor (eCNN) that combines ERNet with FBISA in a more adaptable and scalable design. They demonstrated that ERNet has superior quality, resolution, and de-noising of up to 4K Ultra-HD 30 frames per second while using DDR-400 and 6.94W (Xiong et al., 2019).

Because of the extensive usage of CNN in a variety of disciplines, it is critical to increased throughput and efficiency. To attain these goals, hardware acceleration is extensively researched in academic and industrial settings. Most of the time, several accelerators are utilized to improve throughput and energy economy while speeding up CNN training. One of the difficulties in these methods is determining the optimal way to offer computation and data transfer across multiple accelerators. The authors suggested HyPar as the best approach for predicting layer-wise parallelism in deep networks by using an array of accelerators. The suggested method divides the input and output feature maps, gradient tensors, error tensors, and kernel tensors. The optimization task's main goal is to determine the optimum feasible partition to maximize throughput while minimizing communication costs across accelerators. The authors used a communication model to calculate the cost of communication. Hierarchical layer-wise dynamic programming is used to estimate the partitioning for each layer. The suggested approach has linear time complexity. The suggested model is tested on 10 CNN models including LeNet5, and VGG 19. They determined that model parallelism performs badly, while data parallelism is not always the best. Hybrid-parallelism can outperform both. According to their findings, HyPar increases the performance by 3.39X and has an efficiency gain of 1.51X over default data parallelism. It also has a performance gain of 2.40X over one weird trick (Szegedy et al., 2017).

Python's Ray Library, an innovative inherent library supplied by Python, is utilized to parallelize CPU cores for plant disease detection (Datta et al., 2020). The authors compared research utilizing several deep learning architectures such as AlexNet, VGG16, and Umarex. The authors' comparison analysis revealed that training CNN for image classification on parallelized CPU cores significantly reduced computation time. Because of their computationally expensive processing layers, deep learning architectures pose a significant challenge in practical implementation (Datta et al., 2020).

Facial expression recognition is a crucial and active research issue in computer vision (Deb et al., 2020). The authors employed two benchmark parallel CNN networks designed for computational speed performance, viz AlexNet and VGG16. These two network models were employed to extract features before SVM is utilized to conduct multi-class classification. The authors discovered that AlexNet had an accuracy rate of 86.06% while VGG16 had an accuracy rate of around 80%. Deep models were trained to identify facial expressions, including neutral, smile, surprise, squint, disgust, and scream (Deb et al., 2020).

They developed a strategy for detecting and removing malware, which is in demand for interconnected devices (Bakhshinejad and Hamzeh, 2020). For malware detection, the authors created a unique technique based on CNNs parallel architecture. The technique utilized raw bytes from executable files, eliminating the requirement for extraction of high-level features (Bakhshinejad and Hamzeh, 2020).

## 4. Motivation

Due to the remarkable success of deep learning techniques in various fields such as computer vision, satellite imagery, medical image analysis, and cosmology, training acceleration of CNNs is of utmost importance and has become a crucial problem. At present high-performance computing (HPC) configured with high-end CPUs and GPUs are utilized to accelerate CNN training, however, they still cannot meet the computational, memory, and energy requirements. It is a hot research topic for those organizations having an ample amount of data for training. Google has also issued the latest edition of the TPU (Strubell et al., 2019) after its 1st edition (Jouppi et al., 2017) for the maximization of CNN inference. A lot of research is ongoing on the acceleration of DNN training; some researchers consider the accelerator, which only focused on maximizing inference and not on CNN training. Only some of the existing accelerators (Kim et al., 2016; Song et al., 2017) are taking into consideration CNN inference as well as DNN training acceleration. Current accelerators such as Neuro-cube (Kim et al., 2016) perform partitioning of models among HMC vaults, but parallelism between HMC is not considered. Pipe layer (Song et al., 2017) explored the concept of intra-layer parallelism to enhance training performance, but data movements for both inter-layer and intra-layer parallelism are still to be explored.

To meet the expectation of high throughput and high energy efficiency, CNN training acceleration models (Simonyan and Zisserman, 2014; He et al., 2016) and with ample amount of data needs exploration of coarse grain parallelism rather than fine-grain parallelism within a layer. Existing solutions cannot completely resolve the problem, which is still an open problem (Chen et al., 2014a,b, 2016; Lu et al., 2017). They only consider intra-layer parallelism and assume data is present in memory already. The assumption is accepted for standalone accelerator processing of an individual layer, as the focus is only on fine-grained computation within a layer. Recent research trends and efforts on hybrid or mixed parallelism (Krizhevsky, 2014; Song et al., 2017; Wang et al., 2018) and layer-wise parallelism motivate us to develop a solution for processing massive amounts of data with deeper models. This can be achieved by exploration of layer-wise or mixed parallelism to increase its computation time without compromising the accuracy, which is the most important parameter in bio-medical image analysis.

## 5. Proposed methodology, optimization techniques employed

### 5.1. Novel activation function

The activation function is taken from the derivation of the diffusion equation based upon some essential properties such as normalized and unsaturated functions showing improvement over the popular ReLu activation function. It helps fix the dying ReLu problem, and being normalized and balanced makes learning faster than already existing activation functions. The activation function is normalized, balanced, and centerd at 0, which makes optimization faster, unlike the sigmoid function, whose curve is S-shaped but not centerd at 0, which makes optimization harder. The function takes both negative as well as positive values. The mathematical formula of the proposed activation function is given by Equation (1), and the first and second order derivatives of the proposed activation function are given by Equations (2) and (3), respectively.


(1) $$\begin{array}{l} \mu_{\alpha }\left(x\right)=\frac{1}{2\sqrt{\alpha \pi }}\exp\left(-\frac{x^{2}}{4\alpha }\right)\;,\;-\infty <x<\infty \end{array}$$

(2) $$\begin{array}{l} \mu_{\alpha }^{\prime }\left(x\right)=-\frac{x}{4\alpha \sqrt{\alpha \pi }}\exp\left(-\frac{x^{2}}{4\alpha }\right)\;,\;-\infty <x<\infty \end{array}$$

(3) $$\begin{array}{l} \mu_{\alpha }^{\prime \prime }\left(x\right)=\frac{-x^{2}+2\alpha }{8\alpha^{2}\sqrt{\alpha \pi }}\exp\left(-\frac{x^{2}}{4\alpha }\right)\;,\;-\infty <x<\infty \end{array}$$

**Figure 2:**
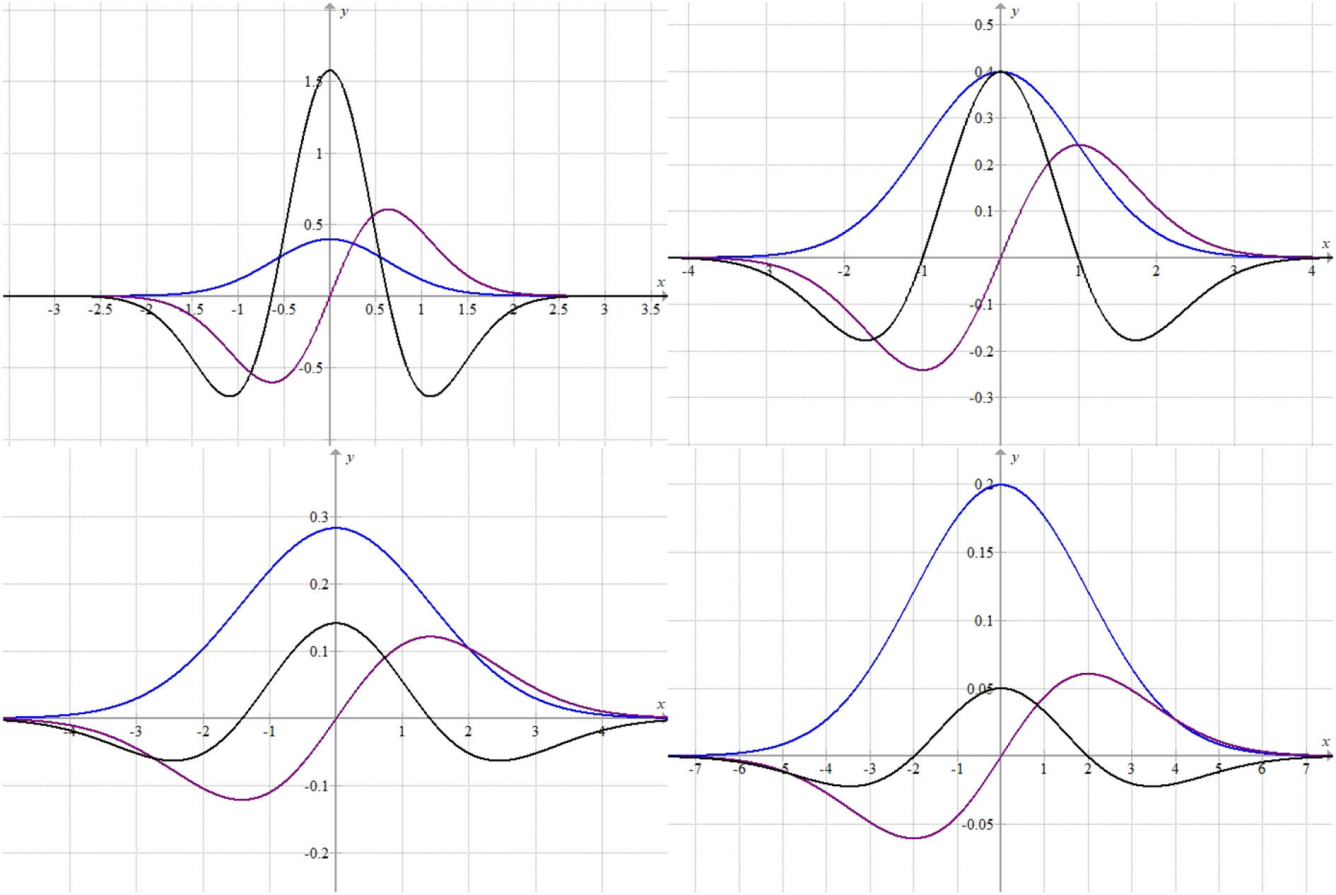
Plots of activation functions for different α's.

On varying standard deviation (α) of the data, we got a family of graphs, for example, for α = 0.2, 0.5, 1, 2 the respective graphs obtained are plotted in Figure 2.

### 5.2. Data augmentation

**Figure 3:**
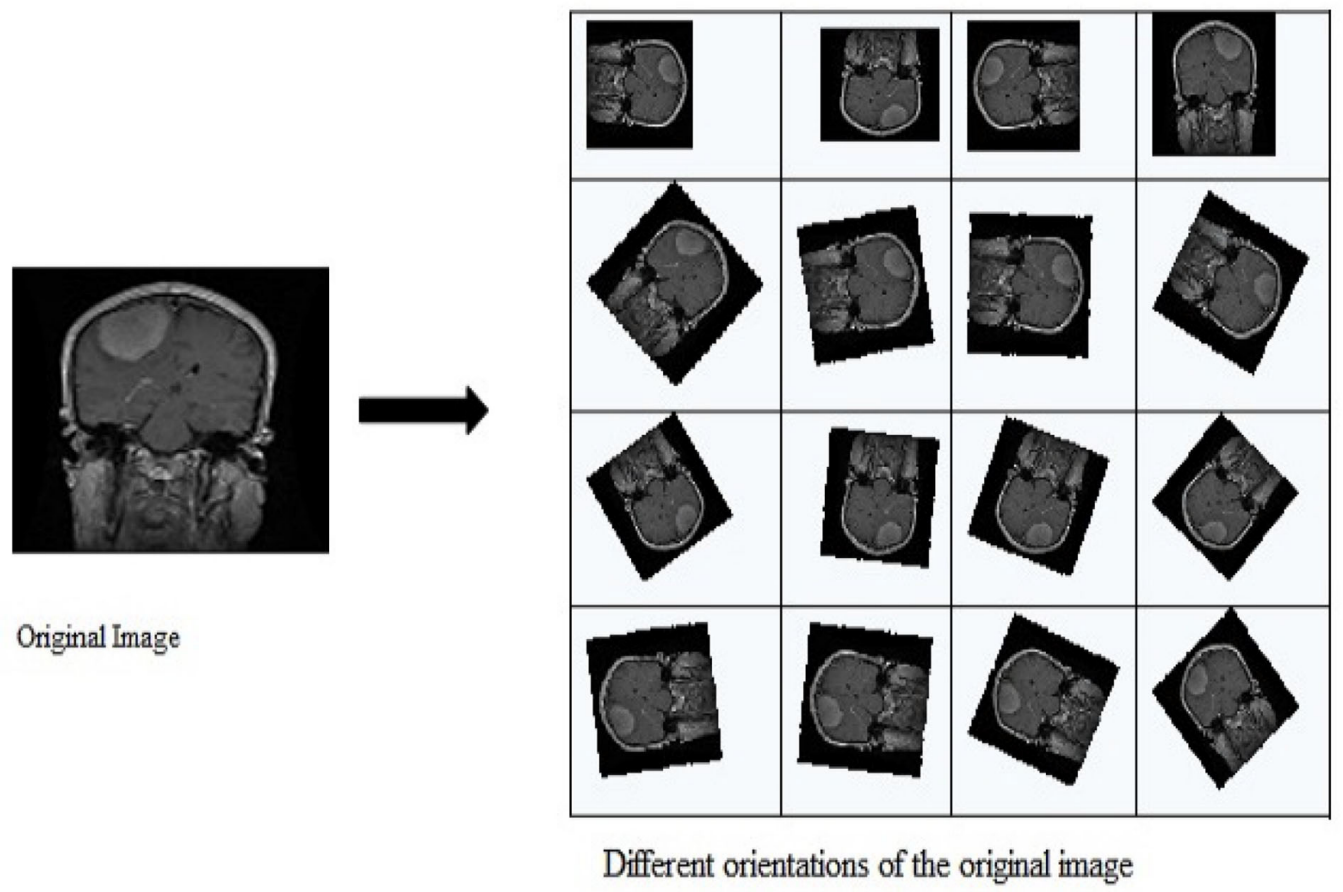
Image with different orientations.

Data Augmentation involves generating more training data when insufficient data is available. This generates data by applying transformations such as color jittering, translation, rotation, and change of orientation by using different angles. It is known that CNN robustly detects and classifies objects, even if aligned in various orientations, an important property of CNNs called translational invariance. CNN is invariant to translations, viewpoints, size, or illumination. This forms the basis for data augmentation. Sometimes, a limited dataset may be available containing images taken in a restrained environment. But our application may have numerous conditions such as scale, illumination, and location. In such situations, CNN is trained with additional synthetically modified data. Figure 3 illustrates how different images can be generated from a single image by performing rotations at different angles.

### 5.3. Transfer learning

The concept behind transfer learning is simply that the model has already learned generic features from a large ImageNet dataset. It can be a generic model for computer vision tasks. There are two ways to customize our pre-trained model, either by using feature extraction or by using fine-tuning. Simply, transferring features from pre-trained models trained on upstream tasks, then fine-tuning the model on downstream tasks is the basic concept behind transfer learning. This actually saves the inference time compared to training from scratch.

#### 5.3.1. Feature extraction

It utilizes generic features already learned by the pre-trained model to extract more specific features from the unseen data sample.

#### 5.3.2. Fine-tuning

The model's initial layers are frozen, and the top layers of this frozen model are trained in conjunction with recently added classifier layers and the base model's final layers. This allows us to fine-tune the higher-order features of the original model by making it more suitable for certain applications.

#### 5.3.3. Global Average Pooling

The main motive behind adding Global Average Pooling (GAP) to the base model is that the former computes the average output of each feature map of the proceeding layer and makes the model ready for the final classification layer. This layer greatly helps in reducing the data and does not contain any trainable parameters such as Max Pooling. GAP helps in the stabilization of validation accuracy, which is a sign of overfitting. Thus, GAP in association with the base model helps reduce the overfitting of the model and the overall computation time of the CNN model.

#### 5.3.4. Regularization technique (dropout)

In association with L2 and L1 regularization, dropout is another popular and powerful regularization technique employed in our proposed CNN model. Dropout simply turns off some neurons with some probability P during training of CNN. Usually, a dropout of 0.5 or 0.25 is used in the majority of CNNs. When *P* = 0.5, half of the neurons are inactive and not considered a part of CNN. *P* = 0.25 means 25% of neurons are not active. With the implementation of dropout, the neural network's complexity becomes less and helps reduce the over-fitting of the CNN model.

### 5.4. The architecture of the proposed model

**Figure 4:**
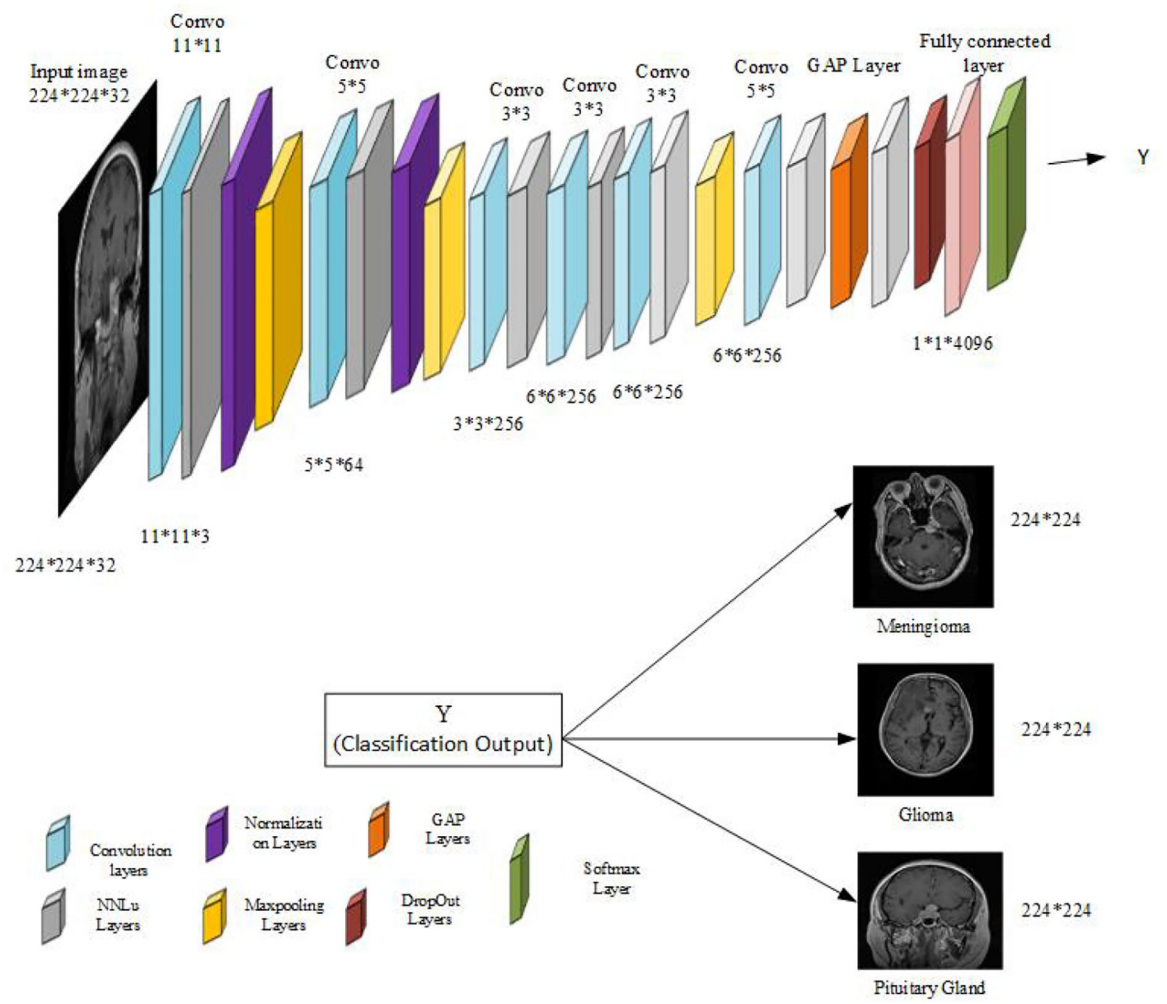
CNN model with GAP layer and hybrid parallelism.

The architecture of the intended model based on AlexNet [33] is given in Figure 4. The model consists of one 11 × 11, two 5 × 5, and three 3 × 3 Conv layers, two normalization layers, eight ReLU layers, and three fully connected layers, with one fully connected layer replaced by the Global Average Pooling layer (GAP). The model consists of the proposed diffusion based non-linear activation instead of ReLu, very similar to the ReLu non-saturated activation layer followed by every convolutional layer in the proposed model. The model consists of one dropout layer to avoid over-fitting and one softmax layer to output the probability distribution. Input images of dimensions 224 × 224 × 32 are fed to the model for end-to-end classification. The model performs multi-classification and provides the corresponding category such as glioma, meningioma, and pituitary tumor. The introduction of the GAP layer in the model considerably improves generalization performance and helps in the reduction of model over-fitting. Also, it enhances the overall computational performance of the model. After the introduction of the novel activation function and GAP layer, the classification accuracy and training time improve significantly.

**Figure 5:**
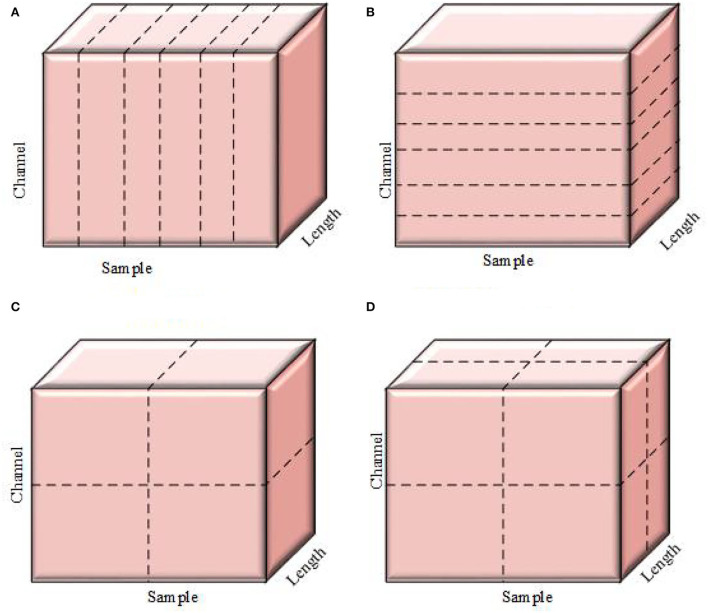
CNN parallelization techniques. **(A)** Data parallelism. **(B)** Model parallelism. **(C)** Hybrid parallelism (S,P). **(D)** Hybrid parallelism (S,A,P). S, sample; A, attribute; P, parameter parallelism.

The Conv layer performs a considerable number of computations, therefore, it is the layer that requires the most processing power. Input to the fully connected layer is actually output coming from the pooling layer or Conv layer and is again fed to the fully connected layer. Thus, the fully connected layer is responsible for generating output or performing flattening, demanding huge memory requirements. These computational and memory-intensive constraints of the convolutional layer and fully connected layer make the practical implementation of the CNN cumbersome, particularly in real-time applications.To mitigate such issues, parallelism is of utmost importance to reduce computational costs. Most of the time, researchers either employ data parallelism or model parallelism. Model parallelism performs worst, and data parallelism cannot be regarded as best. Thus, we prefer an amalgam of data and model parallelism known as hybrid parallelism (HyPar). HyPar implements both data parallelism at the Conv layers and model parallelism at the full connected layers to improve the CNN model's overall efficiency and reduce the overall memory cost. A high-level view of all three types of parallelization techniques is given in Figure 5. Data parallelism performs vertical partitioning, and model parallelism performs horizontal partitioning. Hybrid parallelism performs both horizontal as well as vertical partitioning across either sample and parameter, or sample attribute and parameter.

### 5.5. Feature extraction

**Figure 6:**
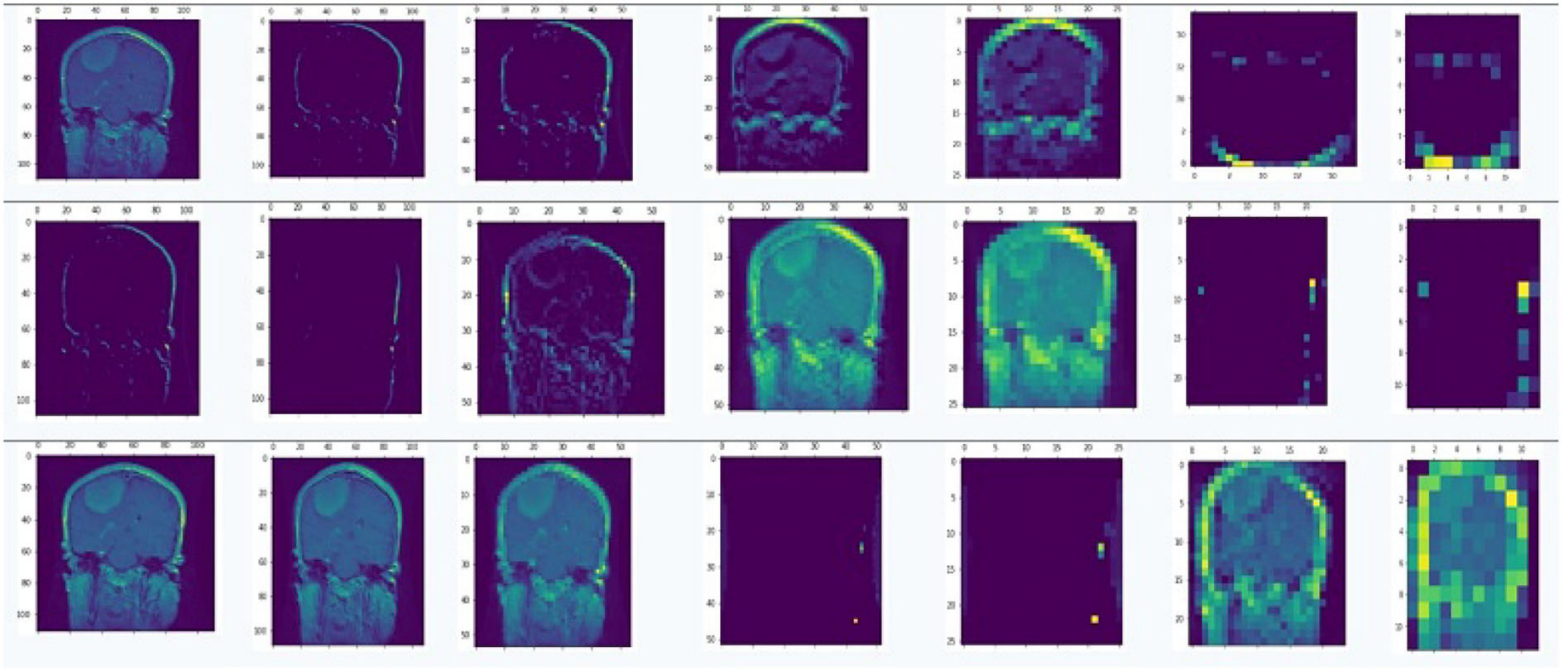
Feature extraction at different convolutional layers using different kernels.

The convolutional layer performs the automatic feature extraction. Generic features like edges, curves, lines, and contours are inherited by the ImageNet database using transfer learning. The initial layers of the model are frozen, and these inherited generic feature vectors are utilized. The hyperparameter tuning for the rest of the layers of the CNN model is done in order to perform the specific feature extraction. More specific features are extracted from higher conv layers. The snapshot of the features extracted at different Convo layers is given in Figure 6. The figure shows clearly how the extracted features are visible at lower convolutional layers. Upon reaching higher convolutional layers, the features become more abstract and are not distinguishable.

### 5.6. Implementation details and experimental evaluation

The dataset used for experimental evaluation consists of a total of 30640 T1 weighted contrast-enhanced images collected from 233 patients with three categories of tumors: glioma, meningioma, and pituitary tumor. It consists of 7,080 slices of meningioma, 14,260 slices of glioma, and 9,300 slices of pituitary tumor. Pre-trained VGG-19 model is used for experimental evaluation and classification of three types of tumors. In this study, Amazon AWS with corresponding CPU instances is used. The machine's configuration for implementing hybrid parallelization is given as 8 high-frequency Intel Xenon E5-2670 processors, NVIDIA Grid K520 CPU with 1536 CUDA cores, and 4GB of video memory. The supporting libraries such as CUDNN 7.4.1, GCC 7.3.1 spectrum, and MPI 2019.01.30 are used for the message passing system during communication of GPUs and NCCL 2.4.2. The rest of the experiments are done using Google Colab with supporting GPU and TPU configurations.

The proposed study can be applied to any deeper neural network such as VGG-16, Resnet-50, and Resnet-XT, and any dataset can be used to verify the observations. For simplicity, we have implemented a neural network model based on a pre-trained VGG-19 network fine-tuned as our requirement. We have used a large-scale brain tumor dataset for experimental evaluation. Our main motive in this study is to optimize the network to have improved computation time for the task mentioned above without degrading the accuracy. Model parallelism outperforms using many neurons, and data-parallelism outperforms in the case of a large number of weights. In CNN, about 90% of computations are performed with convolutional layers and consist of only 5% of the parameters. In contrast, a fully connected layer consists of 95% of the parameters and is responsible for 5–10% of the computations. Therefore, the better choice is to use data-model parallelism in CNN. The algorithm for data parallelism for Conv layers, and model parallelism for fully connected layers is given as:

**Algorithm for data parallelism**


  Data parallel SGD (parameters, data, n) do 
  Distribute data on all available nodes, so every 
  node holds its subset of the dataset. 
  For each node, *I*∈(1, 2, 3, *n*) in parallel, do 
  *k*_*i*_← SGD(parameters, data) 
  Aggregate gradients from all nodes *k*, as
  $\left(\frac{1}{n}\overset{n}{\underset{j=1}{\sum }}\left[k_{j}\right]\right)$ 
  Return *k* 
  end 


The validity of data parallelism can be verified mathematically as:


$$\begin{array}{l} \frac{\partial \text{Loss}}{\partial \omega }=\frac{\partial \left[\frac{1}{n}\sum_{i=1}^{n}g(u_{i},v_{i})\right]}{\partial \omega }=\frac{1}{n}\sum_{i=1}^{n}\frac{\partial g(u_{i},v_{i})}{\partial \omega }\\ =\frac{m_{1}}{n}\frac{\partial \left[\frac{1}{m_{1}}\sum_{i=1}^{m_{1}}g(u_{i},v_{i})\right]}{\partial \omega }+\cdots \\ +\frac{m_{k}}{n}\frac{\partial \left[\frac{1}{m_{k}}\sum_{i=m_{k-1}+1}^{m_{k-1}+m_{k}}g(u_{i},v_{i})\right]}{\partial \omega }\\ =\frac{m_{1}}{n}\frac{\partial p_{1}}{\partial \omega }+\cdots +\frac{m_{k}}{n}\frac{\partial p_{k}}{\partial \omega } \end{array}$$

Where, *ω* represents a hyperparameter, $\frac{\partial \text{Loss}}{\partial \omega }$ is true gradient batch size *n*, $\frac{\partial p_{k}}{\partial \omega }$ is the gradient of the small batch in GPU node *k*, *u*_*i*_, *v*_*i*_ represent features and labels of data point *i*, *g*(*u*_*i*_, *v*_*i*_) is loss for data point *i* calculated from the forward propagation, *n* total number of data points, *k* total number of GPUs nodes, *m*_*k*_ is the number of data points assigned to GPU/node *k*, *m*_1_ + *m*_2_ + ⋯ + *m*_*k*_ = *n*. When $m_{1}=m_{2}=\cdots =m_{k}=\frac{n}{k}$ than


(4) $$\begin{array}{l} \frac{\partial \text{Loss}}{\partial \omega }=\frac{1}{k}\left[\frac{\partial p_{1}}{\partial \omega }+\frac{\partial p_{2}}{\partial \omega }+\cdots +\frac{\partial p_{k}}{\partial \omega }\right] \end{array}$$

### 5.7. Model parallelism for fully connected layers

The algorithm for this approach is given by:


  Model parallel SGD (parameters, data, n) do 
  Partition the kernels, feature maps or layers 
  across GPU nodes and assigning them to devices 
  Implement an automatic movement of tensors 
  between GPUs whenever required 
  Automatically pipeline the execution strategy to 
  take full advantage of all the GPU's. 
  end


**Figure 7:**
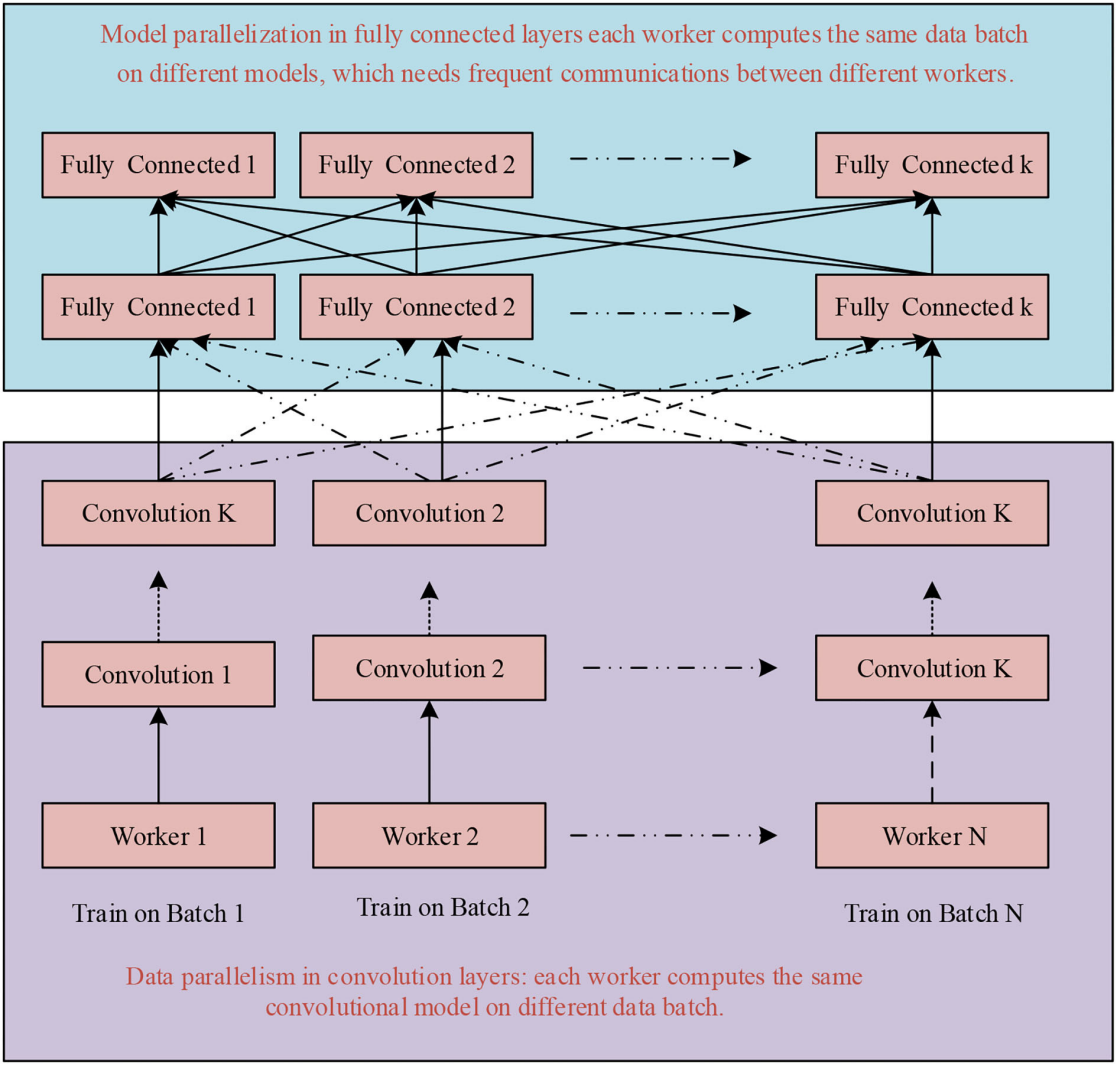
Hybrid parallelism.

The illustration of the implementation of hybrid parallelism for both Conv and fully connected layers is shown in Figure 7.

The proposed network parameters are given in Table 1. We have used a stride of 1 for Conv layers and a stride of 2 for pooling layers. Also, padding of width 1 is used for all the layers. Bias =0.1, weight initialization= Xavier, α = 0.333, drop out= 0.5, batch size = 128, and default optimizer =Adam.

**Table 1:** Hyperparameters of the proposed model.

**Layers**	**Kernel size**	**No of filters**	**Output calculation**
Conv 1	11 × 11 × 3	64	224 × 24 × 64
Pool 1	3 × 3	2	112 × 112 × 64
Conv 2	5 × 5 × 64 Stride = 2	256	112 × 112 × 256
Pool 2	3 × 3	2	56 × 56 × 256
Conv 3	3 × 3 × 256	256	56 × 56 × 256
Conv 4	3 × 3 × 256	256	56 × 56 × 256
Conv 5	6 × 6 × 256	4,096	56 × 56 × 4096
Pool 3	2 × 2	2	28 × 28 × 4096
Conv 6	6 × 6 × 256	4,096	28 × 28 × 4096
Pool 4	2 × 2	2	14 × 14 × 4096
Fc 1	1 × 1 × 4096	4,096	4,096
Global Average Pooling layer			
Fc 2	1 × 1 × 4096	3	4,096

## 6. Results and discussions

**Figure 8:**
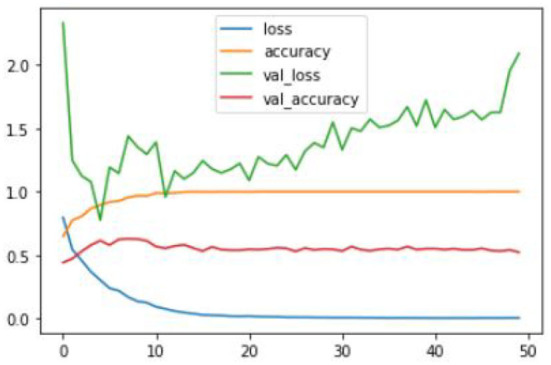
Proposed CNN training graph.

**Figure 9:**
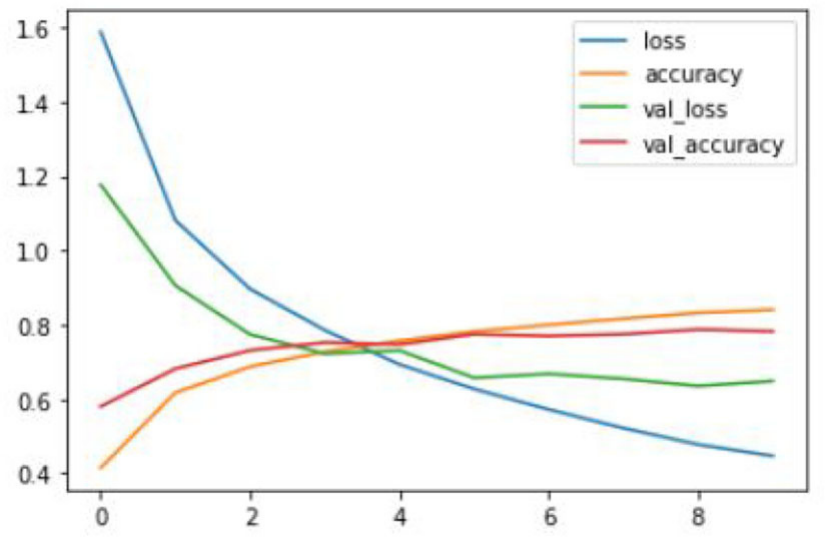
Proposed CNN+Novel NNLU training graph.

**Figure 10:**
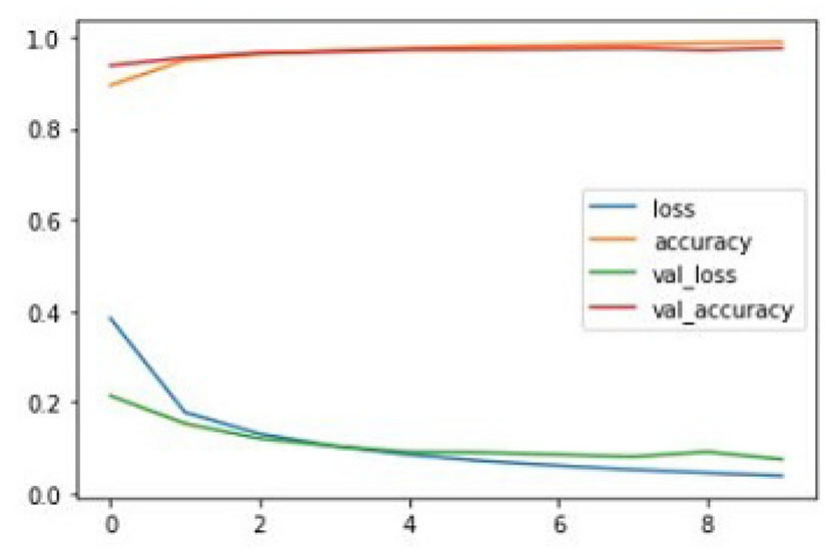
Proposed CNN+Novel NNLU+GAP layer training graph.

After training the proposed CNN model with given ground truths, the former can classify the given dataset into three types of tumors: glioma, meningioma, and pituitary tumor. The training graphs of the proposed model with the loss curves, accuracy curves, and validation loss and accuracy are given in Figures 8–10.

The proposed model outperforms others in classification accuracy and provides a classification accuracy of 98.89% when both NNLU and GAP layers are used. It also has a classification rate of 84% for end-to-end classification without introducing NNLU and the GAP layer. The training time of the basic proposed model is 18 mins and 5 s, and when the GAP layer and transfer learning are used, the training time reduces to 3 s. GAP and NNLU help in reducing the over-fitting of the model and improve the classification accuracy rate compared with existing methods (Habib and Qureshi, 2020a).

**Figure 11:**
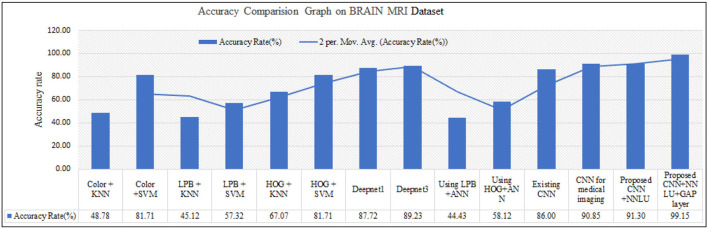
Proposed method classification accuracy and comparison with existing methods.

**Figure 12:**
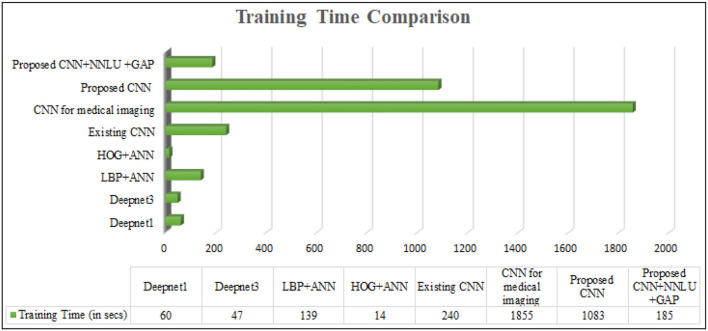
Time complexity comparison.

The overall performance metric is recorded and compared with existing methods. The method is observed to outperform others in classification accuracy as given in Figure 11. The computational speed of the proposed method is recorded and can also be seen in Figure 12. The proposed method is compared with existing methods (Habib and Qureshi, 2020a) and outperforms them.

**Figure 13:**
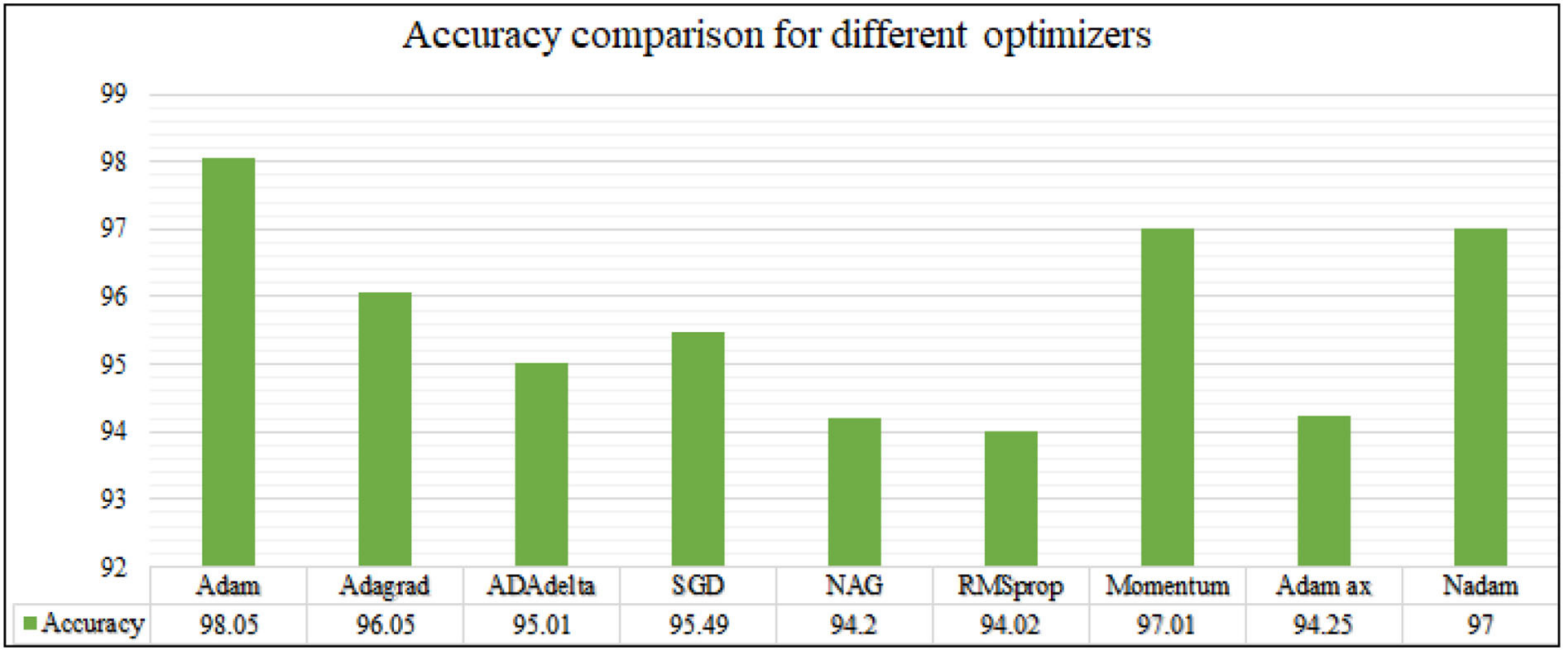
For epoch 30.

**Figure 14:**
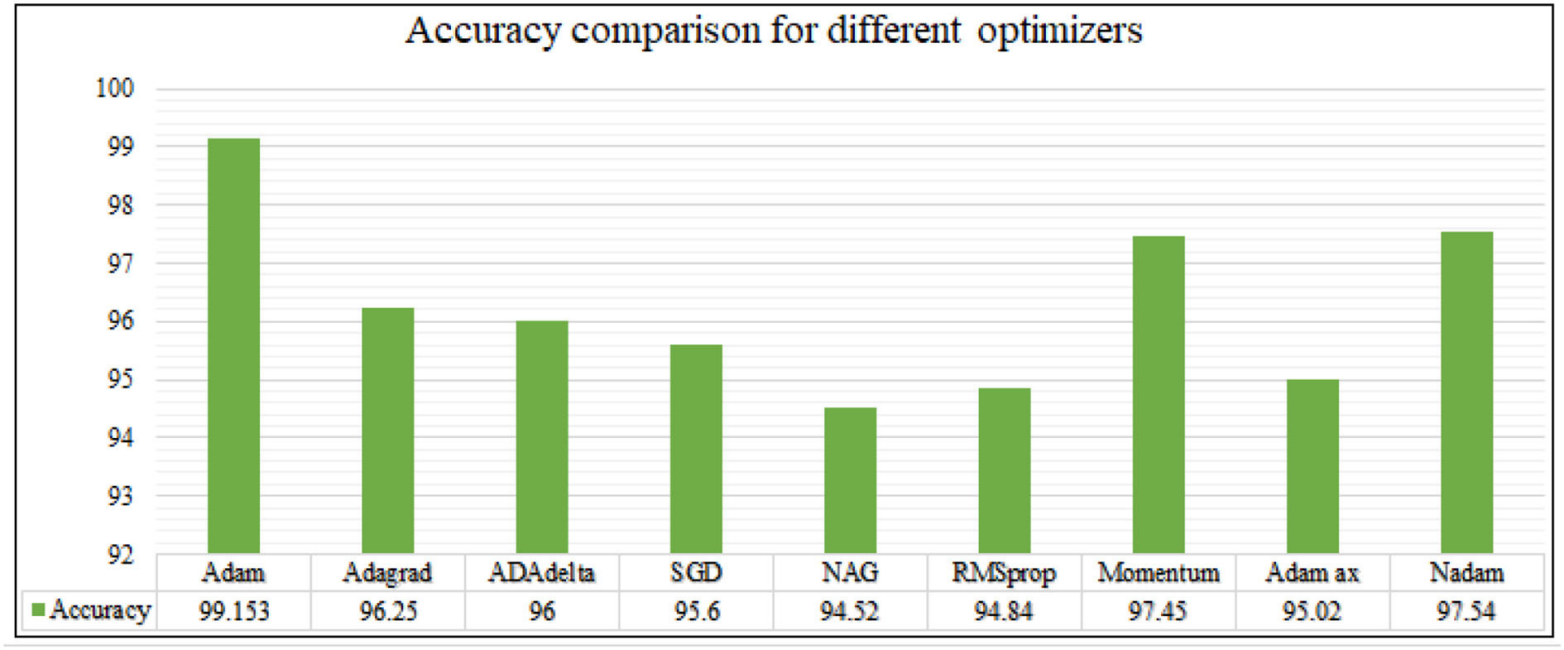
For epoch 50.

The analysis of training accuracy is further done for various optimization methods, and the accuracy is observed for different epochs. The results are recorded and shown as in Figures 13, 14.

The major limitation of the existing state of art methods is that although they perform well in terms of classification accuracy, they lag as far as computational speed is concerned. To mitigate the issue, hybrid parallelization is implemented with the proposed model to improve the computational speed. The improved computational speed with the proposed parallelization strategy is achieved by using four GPU nodes with varying batch sizes. Observations are given in Table 2.

**Table 2:** Speedup with varying batch sizes and cross entropies.

**No. of nodes**	**Batch size (*m, n*)**	**Cross entropy**	**Speed up**	**Training time in seconds (s)**
1	(56, 56)	2.521	1.2X	122
2	(128, 128)	2.524	1.53X	184
2	(128, 56)	2.523	3.59X	65
3	(512, 512)	2.614	3.60X	60
3	(512, 128)	2.645	3.62X	15

Here, m and n denote the effective batch sizes in convolutional layers and the fully connected layers, respectively. Model accuracy is given when various parallelization techniques are employed. When one epoch of training is computed on multiple GPUs, it is observed that the training time is reduced due to better utilization of hardware resources. Also, the performance analysis of various parallelization techniques is given in Table 3. The results in the table are recorded by varying alpha and keeping the number of nodes as 2.

**Table 3:** Performance of proposed Model+NNLU+GAP layer.

**Parallelization scheme**	**Number of nodes**	**α**	**Min. training loss**	**Min. val. loss**	**Max. val. accuracy (%)**
Data parallelization	2	0.0001	0.2030161	0.7956214	81.5732
Model parallelization	2	0.0002	0.2023145	0.8241562	81.052
Hybrid parallelization	2	0.0005	0.20002365	0.815673	97.37

**Figure 15:**
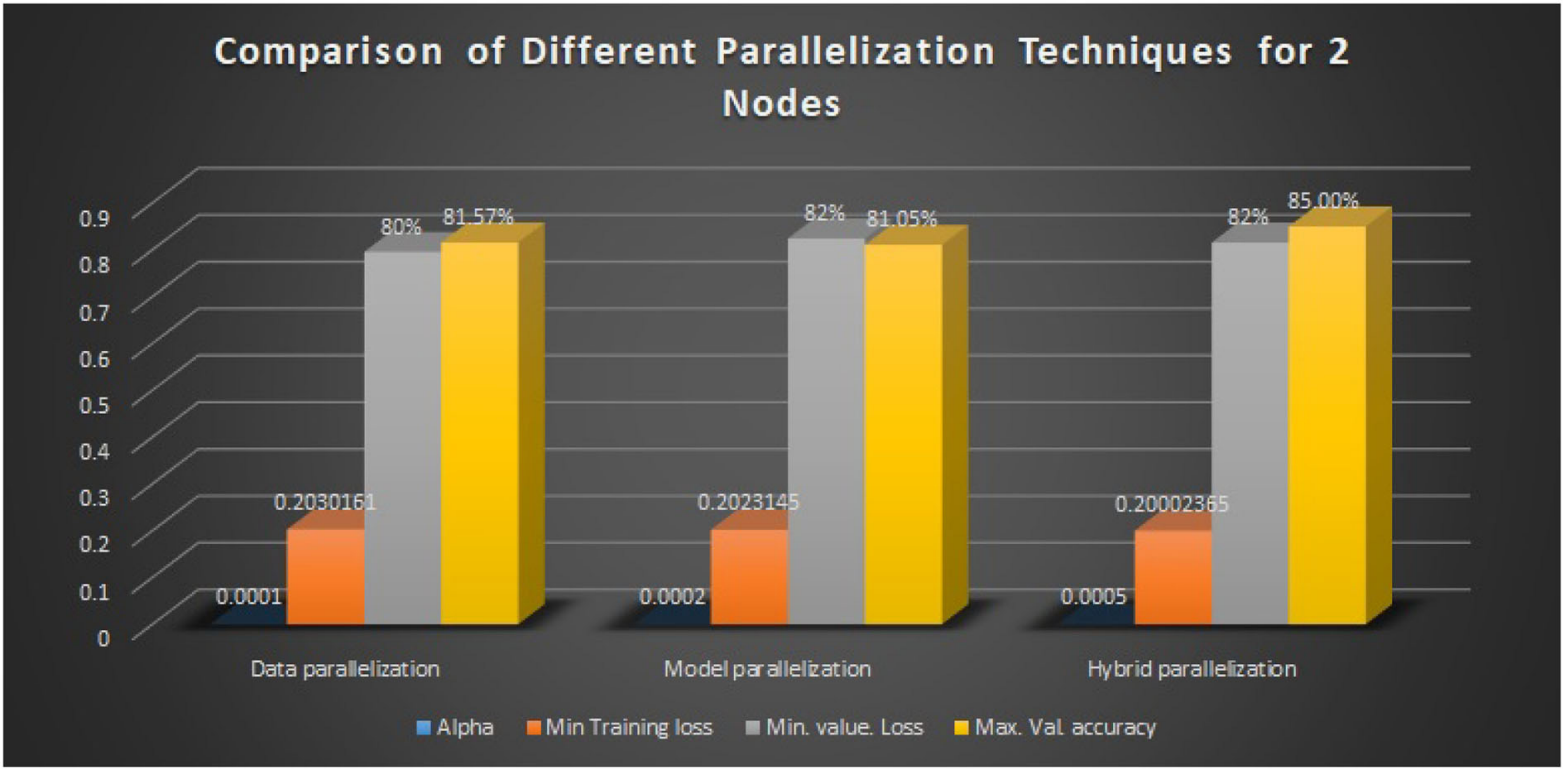
Performance metric comparison graph.

The graphical visualization is given in Figure 15 below.

After applying parallelization techniques, the computational time and training speed are improved due to efficient hardware resource utilization, with an accuracy drop of 1.5%.

### 6.1. Empirical analysis

#### 6.1.1. Computation cost

SGD is used on each machine, achieving an error of less than ϵ. The computation time of SGD on every individual machine is given as: $O\left(P\log\frac{1}{\epsilon }\right)$

where *P* denotes the parameter size and $O\left(P\log\frac{1}{\epsilon }\right)$ denotes the number of iterations required for convergence.

The computation time required for the aggregate operation of gradients is:


(5) $$\begin{array}{l} \text{Total number of communication rounds}=\log k \end{array}$$

In each epoch summation of parameters is done, which is *S* = *O*(*P*) on a single processor.

With *N* number of Processors $\text{Sum}=O\left(\frac{P}{N}+\log k\right)$


(6) $$\begin{array}{l} \text{Computation time}=O\left(\frac{P}{N}+\log k\right)+O\left(P\log\frac{1}{\epsilon }\right) \end{array}$$

#### 6.1.2. Communication cost

SGD is locally calculated on each machine, so it incurs no communication cost for SGD. As every individual machine updates its parameters locally, all in one communication is performed to send the updated parameters to the parameter server, where the average is computed.


(7) $$\begin{array}{l} \text{Communication Cost}=L\left(\frac{k}{2}+\frac{k}{4}+\cdots \right)\\ +\frac{kP}{B}\left(\frac{1}{2}+\frac{1}{4}+\frac{1}{8}+\cdots \right) \end{array}$$

(8) $$\begin{array}{l} =O\left(Lk\right)+O\left(\frac{kP}{B}\right) \end{array}$$

For *N* number of parameter servers, the total Communication cost is given by *O*(*Nk*)+*O*(*Pk*).


(9) $$\begin{array}{l} \text{Total Communication time}=O\left(kN\right)=O\left(N\right) \end{array}$$

where $N=\frac{D}{K}$.

Finally, the proposed method is compared with the state-of-the-art methods in terms of computation time, communication cost, and communication time and are summarized in Table 4 as:

**Table 4:** Time complexity and communication cost comparison.

**Proposed algorithm**	**Existing algorithm**
$\text{Computation time}=O\left(\frac{P}{N}+\log k\right)+O\left(P\log\frac{1}{\epsilon }\right)$	$\text{Computation time}=O\left(P\log k\log\frac{1}{\epsilon }\right)+O\left(P\log\frac{1}{\epsilon }\right)$
Communication cost = *O*(*Nk*)+*O*(*Pk*)	$\text{Communication cost}=O\left(Pk\log\frac{1}{\epsilon }\right)$
Communication time = *O*(*N*)	$\text{Communication time}=O\left(P\log\frac{1}{\epsilon }\log k\right)$

From the comparison table, it is clear that the computation time and the communication time are better in our proposed technique than in other state-of-the-art methods. Computation power can be further improved by using more powerful models such as vision transformers (Han et al., 2022; Shin et al., 2022). Besides adding more computational power, we can even get better models for real time applications with constrained resource requirements by having a closer look at the compression techniques (Chen, 2022). The attention mechanism in CNNs can improve further computation power of the proposed model (Guo et al., 2022).

## 7. Conclusion, limitations, and future scope

In this study, a novel activation function is proposed together with a new hybrid parallelization technique. We present a CNN model in which the Global Average Pooling layers entirely replace one of the fully connected layers. This helps in overcoming the over-fitting problem in the network and also improves the training time and classification accuracy. Also, to speed up the proposed network, a hybrid parallelization strategy is implemented that significantly reduces the training time and improves the computation speed by 4.73X when a batch size of (512,128) is used. A slight accuracy drop of 1.5% is observed. Experimental results show that data and model parallelism perform almost the same. Hybrid parallelism outperforms both of these and yields a validation accuracy of approximately 85% with minimum training loss.

CNNs have achieved remarkable success when applied to computer vision tasks due to strong inductive bias. But at the same time, they consume not only large computational resources but also a lot of memory resources. That limits their implementation in practical applications such as embedded devices and edge devices. CNNs are less computationally efficient as compared to vision transformers. In future CNNs,we intend to replace them with these more powerful models. In the future, CNNs can be replaced by these powerful models. Also, in the future, we will pay more attention to the compression of CNNs and will make them compressed and sparse, which makes their application more suitable for resource constrained devices. The introduction of an attention mechanism in CNNs can also make them more powerful for computer vision tasks.

## Data availability statement

The datasets presented in this study can be found in online repositories. The names of the repository/repositories and accession number(s) can be found below: https://figshare.com/articles/dataset/brain_tumor_dataset/1512427.

## Ethics statement

Written informed consent was not obtained from the individual(s) for the publication of any potentially identifiable images or data included in this article.

## Author contributions

Both authors involved in full contribution of the research work. Both authors contributed to the article and approved the submitted version.

## Conflict of interest

The authors declare that the research was conducted in the absence of any commercial or financial relationships that could be construed as a potential conflict of interest.

## Publisher's note

All claims expressed in this article are solely those of the authors and do not necessarily represent those of their affiliated organizations, or those of the publisher, the editors and the reviewers. Any product that may be evaluated in this article, or claim that may be made by its manufacturer, is not guaranteed or endorsed by the publisher.
